# Interspecific Competition for Shelters in Territorial and Gregarious Intertidal Grazers: Consequences for Individual Behaviour

**DOI:** 10.1371/journal.pone.0046205

**Published:** 2012-09-25

**Authors:** Moisés A. Aguilera, Sergio A. Navarrete

**Affiliations:** 1 Centro de Estudios Avanzados en Zonas Áridas (CEAZA), Facultad de Ciencias del Mar, Universidad Católica del Norte, Coquimbo, Chile; 2 Estación Costera de Investigaciones Marinas, Las Cruces, and Center for Marine Conservation, Pontificia Universidad Católica de Chile, Santiago, Chile; University College Dublin, Ireland

## Abstract

Experiments have shown that interspecific interactions within consumer guilds can alter patterns of distribution, abundance and size of species. Plastic behavioural responses can be modulated by agonistic interactions. In many cases, consumers compete for space and shelters, and these interactions change the manner in which they exploit food. This study investigates the consequences of competition in the spatial and temporal organization of behaviour of intertidal grazers, which share algal resources and the use of rock crevices while resting, but exhibit different body sizes, spatial behaviour and foraging modes. We evaluate interaction strength between small gregarious *Siphonaria lessoni* and the larger territorial keyhole limpet *Fissurella crassa* and between *S. lessoni* and the medium-size gregarious chiton *Chiton granosus*. Using field manipulations and artificial arenas in the laboratory, we tested whether the use of crevices, micro-spatial distribution and activity are modified by the density of conspecifics and the presence of heterospecifics. Our results show that small-scale spatial segregation observed in the field between *S. lessoni* and *C. granosus* result from species-specific differences in habitat use. In turn, we found evidence that spatial segregation between *F. crassa* and *S. lessoni* results from highly asymmetric interference competition in the use of shelters. The presence of *F. crassa* reduced the use of crevices and growth rates of *S. lessoni.* Effects on growth rates are assumed to result from exposure to harsh environmental conditions rather than food limitation. Thus, neither gregarious behaviour nor differences in activity were sufficient to prevent competition with the larger grazer. Our study illustrates the importance of competition for shelters, which results in behavioural changes of the smaller-sized species, and how these plastic responses can translate into differences in growth rates. Use of shelters can thus be modulated by environmental conditions in a species-specific as well as an interactive manner within consumers’ guilds.

## Introduction

Determining the structure of interactions among species within guilds of consumers, or within functional groups in general, has become of increasing importance as ecologists attempt to decipher the mechanisms underlying resilience of particular ecosystem functions [1], [2]. For instance, interspecific competition leading to niche partitioning and differentiation of species requirements is one of the mechanisms that can foster complementarity of functions [3], [4], [5]. But competition between coexisting species may also reduce the gain in community-level function expected from resource partitioning [6]. Within diverse consumer guilds, species also share and often compete for non food resources with members of the same or other guilds, resulting in patterns of spatial distribution and/or temporal activity patterns that diminish or enhance the impact of interspecific competition [7], [8], [9]. Thus, both exploitative and interference competition are expected to modify temporal and spatial organization of foraging within guilds (e.g. [10], [11]). Similarly, intrinsic differences in individual traits such as growth, foraging behaviour and activity patterns, can be modulated by agonistic interactions within a guild, and the response can largely determine patterns of coexistence among species [12], [13], [14], [15], as well as their functional roles in the ecosystem [16], [17], [18]. Thus, interactions within members of the guild may not only alter patterns of abundance and spatial distribution of other species, but it can alter the expression of individual traits and, therefore, the functional structure of the guild. Here we examine competitive interactions among members of an intertidal herbivore assemblage which overlap widely in diet and whose combined effects change from redundant to more complementary roles as algal succession progresses [17].

Most rocky shores of the world are characterized by the presence of diverse herbivore assemblages that include species of widely different body sizes, morphologies, life histories and habits [19], [20]. Experimental studies have demonstrated that in many cases the spatial distribution of component species can be determined by interspecific competition for food or space [12], [21], [22], [23]. In the case of molluscan herbivores like limpets and chitons, for instance, resting-site fidelity and foraging strategies of individuals respond to direct interference with the dominant species (i.e. limpets, [21], [24]). The use of rock crevices during resting periods is a common strategy used by many herbivores to avoid environmental stress (e.g. wave action, desiccation, heat stress) and/or to reduce the risk of predation [25], [26], [27], [28]. But these topographic features constitute a limiting spatial resource for which individuals compete against con- and heterospecifics. These interactions can expose subordinate species to harsher conditions [27] and may modify the spatial patterns of their food resources [26], [29]. The need to secure a shelter can sometimes lead to changes in the pattern of day-night activity of individuals (e.g. [30], [31]), allowing species to coexist on an otherwise insufficient resource base. Therefore, both spatial and temporal organization of most species within assemblages could be altered by competitive interactions with one or few dominant species thus determining both spatial and temporal niche segregation [7], [21], [32]. Commonly, the largest sized species is the dominant competitor within the guild, especially when interference competition dominates guild structure [33], [34].

In this study we use field and laboratory experiments to quantify intra- and interspecific interactions in three species of intertidal herbivores that overlap amply in diet; the keyhole limpet *Fissurella crassa*, the pulmonate limpet *Siphonaria lessoni* and the chiton *Chiton granosus*. The target species have different body sizes, spatial behaviour (gregarious versus territorial), foraging modes, and diel activity, but they all share the use of rock crevices while resting. The species *S. lessoni* has diurnal activity rhythms instead of the nocturnal activity that characterize the other species [28]. Despite differences in diel activity, which could be expected to reduce heterospecific encounters and therefore the intensity of interspecific competition [32], previous studies showed that *S. lessoni* segregates at small scales (few centimeters) from *C. granosus* and from *F. crassa* [28]. Thus, it appears that heterospecific encounters, probably for the use of crevices, could shape the patterns of spatial distribution among these species. We therefore tested whether spatial and/or temporal niche segregation occurs in this assemblage as a consequence of competition between species with different traits by evaluating: 1) whether intra- and interspecific competition altered spatial patterns of shelter use (i.e. crevices) behaviour, individual spatial distribution and/or activity rhythms, 2) how these behavioural responses may translate into changes in individual growth rates, and 3) whether body size and gregarious behaviour were determinants to the outcome of these interactions. These hypotheses were tested with a series of field experiments conducted in mid-intertidal rocky shore, and laboratory experiments using artificial arenas placed in outdoor aquaria.

## Materials and Methods

### Ethics Statement

All invertebrate manipulation in both field and laboratory were conducted according to relevant national and international guidelines. All necessary permits were obtained for the described field studies which were provided by the chief director of ECIM (Dr. Sergio A. Navarrete, ECIM-PUC). These permits include access to rocky platforms of the marine reserve at ECIM, and experimental manipulations.

### Focal Species and Study Site

The species *Siphonaria lessoni*, a small sized pulmonate limpet, (0.97±0.07 cm average shell length) together with the chiton *Chiton granosus* (5.6±0.37 cm average body length, see Table 1) are the most abundant grazers in mid to high-intertidal levels along the coast of central-northern Chile [28], [29], [35]. Both species have low to moderate site fidelity and highly gregarious patterns of distribution (Table 1), especially while resting inside rock crevices. Aggregations are maintained, although more loosely, while actively foraging, which occurs during daytime hours in the case of *S. lessoni* and night time in the case of *C. granosus* ([28] and see Table 1). Coexisting in this same assemblage is *Fissurella crassa* which is one of the largest intertidal keyhole limpets in the world (7–10 cm shell length). This is a solitary species with remarkably strong homing behaviour (Table 1). Individuals usually maintain a distance from conspecifics while resting inside crevices during daytime hours and the inter-individual distance increases even farther when foraging at night [28]. Thus, the three species use rock crevices and also overlap widely in diet, which consist mostly of microalgae, ephemeral algae, periphyton and small invertebrates [36], [37]. Previous experiments and field observations suggest that interspecific competition for food resources is of lesser importance than interference in these species, partly because of their ample diet and the comparatively high productivity of benthic micro- and macro-algae observed in the Chilean upwelling ecosystem [38], [39]. Despite differences in diel activity, which could be expected to reduce interspecific encounters and therefore the intensity of interspecific competition [32], previous studies showed that *S. lessoni* segregates at small scales (few centimetres) from *C. granosus* and from *F. crassa* [28]. Thus, it appears that interspecific encounters, probably for the use of crevices, could shape the patterns of spatial distribution among these species.

**Table 1:** Main behavioural and morphological characteristics of the study grazer species.

Grazer species	Individual spatial patterns	Activity phase	Homing behaviour	Displacement lengths when foraging (cm, mean±SE)	Shell size (cm, mean±SE)
*S. lessoni*	Gregarious	Diurnal	Moderate	24.6±3.7	0.97±0.07
*F. crassa*	Dispersive	Nocturnal	Strong	60.1±5.2	6.4±0.72
*C. granosus*	Gregarious	Nocturnal	Moderate	54.2±5.9	5.6*±0.37

* For chitons, it corresponds to body length.

It must be noted that keyhole limpets are heavily exploited by humans, and as a consequence, abundance of larger individuals is low in open access areas [40]. The pulmonate limpet *S. lessoni* and *C. granosus* are not collected by humans. Therefore, field experiments were set up inside the marine reserve of the Estación Costera de Investigaciones Marinas at Las Cruces (hereafter ECIM), in which humans have been excluded since 1982, allowing an increase in abundance and size of *F. crassa* [40].

To quantify the strength and direction of competition for shelters between *F. crassa* and *S. lessoni* and between *S. lessoni* and *C. granosus* we conducted both field and laboratory experiments. Unfortunately, field experiments involving chitons and pulmonate limpets were destroyed by waves two times. Therefore, in addition to short-term laboratory observations, we conducted a longer-term laboratory experiment to evaluate competitive interactions between chitons and pulmonate limpets.

Surveys of grazers were simultaneously conducted at ECIM and Las Cruces, an unprotected area adjacent to ECIM where large limpets are harvested. Natural densities of *S. lessoni* at Las Cruces average 68.9 indiv./m^2^ while densities inside ECIM reach 248.0 indiv./m^2^. Densities of *F. crassa* at Las Cruces average 1.63 indiv/m^2^ and reach 10.1 indiv./m^2^ in some platforms inside ECIM [40]. Commonly *S. lessoni* individuals tend to have smaller size inside the marine reserve, where *F. crassa* individuals are large and abundant, compared with open access areas where large keyhole limpets are collected by humans (*S. lessoni* shell size: ECIM: 0.79±1.15 cm; Pelancura (open access): 1.16±2.27 cm, see Fig. S1).

### Interspecific Encounters of Grazers in the Laboratory

To characterize behavioural responses to interspecific encounters and aid in the design of competition experiments, we conducted short-term laboratory trials in outdoor aquaria at ECIM. Individuals used in experimental trials were collected from nearby intertidal platforms, their wet weight and shell length recorded, and then marked individually with a bee tag glued onto the shell (Opalith-Zeichenplattchen mit kleben, Chr. Graze, Weinstadt-Endersbach, Germany). We recorded individual responses to heterospecific encounters, counting the number of times an interspecific encounter resulted in individuals: clumping to the rock upon contact, halting displacement (i.e. when animals stop moving inside plots), aggressiveness (pushing), and changes in the angle of orientation. Observations were conducted every 30 min over 3 consecutive days, and the assays were repeated two times during February and March 2009. Behavioural responses were expressed as a fraction over the total number of inter-specific encounters observed. The experimental arenas consisted of a 40 × 40 cm concrete blocks with a crevice (20 cm long × 8.0 cm wide × 4.0 cm deep) carved in the centre to provide shelter for individuals, and which resemble those used by animals in the field (see [28]). Arenas were surrounded with a barrier of antifouling paint and a 10 cm high fence made of coarse plastic mesh to prevent grazers from leaving the blocks. These arenas were placed inside outdoor aquaria with running seawater and air, and tides were simulated by slowly draining and refilling the experimental arenas following the natural tidal cycle. A hole, about 1 mm in diameter, was created in one side of the bottom of crevices in order to allow drainage of crevices when draining of aquaria. Surface temperature in the experimental arenas was measured on the flat surface and inside the crevices using an infrared thermometer. Food was provided *ad libitum* by means of acrylic plates (10×10 cm) on which a thick microalgal mat was allowed to grow in the field for three weeks.

At the beginning of these short-term trials, individuals were introduced simultaneously onto the blocks at 15–20 cm distance from the crevice. We enclosed either 10 *S. lessoni* individuals (shell length size = 0.80±0.02 cm) with 1 *F. crassa* (shell length size  = 6.25 cm ±0.12 cm), 10 *S. lessoni* with 3 *C. granosus* (body length size  = 5.11±0.19 cm) and 1 *F. crassa* with 3 *C. granosus*. Arenas with each individual species alone were also monitored. Individuals were left to acclimatize to arenas for three weeks. We maintained the solitary or gregarious nature of these species and resemble average densities found in the field e.g. Las Cruces; *S. lessoni*: 7.83±1.55 indiv × 900 cm^2^; *F. crassa*: 0.71±1.55 indiv ×900 cm^2^; *C. granosus*: 3.97±0.77 indiv ×900 cm^2^ [28] (see also Fig. S1 for shell size of *S. lessoni* and *F. crassa* found at other localities). Arenas were replicated 3 times and no animals were used in more than one trial.

### Competition Experiments in the Laboratory: *C. granosus* and *S. lessoni*

Since field experiments on competition between chitons and pulmonate limpets were destroyed by waves, we conducted a competition experiment between these species in outdoor aquaria under naturally variable environmental conditions and using the same experimental “arenas” as before. The experiment was initiated on March 03 and terminated on April 01, 2009. We examined responses in activity patterns, spatial distribution and the use of crevices. Due to the short-term nature of the experiment, we could not quantify changes in growth. Individuals were assigned to replicated (n = 3) experimental arenas at two density levels to evaluate intraspecific effects: a) natural density (3 individuals of *C. granosus*, 9 individuals of *S. lessoni*), which represented the average densities observed in the field, and b) high densities (6 individuals of *C. granosus*, 18 of *S. lessoni*), which are occasionally seen in the field over small areas. To evaluate inter-specific effects, we used: c) a mixed species treatment in which individuals of both species were included in the experimental arenas at their average density levels, i.e. 12 individuals per plot. Enclosures of both species at high densities were also attempted, but animals tended to escape from arenas. Intraspecific enclosures at high density were also impossible to maintain for *C. granosus*. Therefore, the design and data analysis differed from that used in previous experiments, where individuals of all species have roughly similar body size and habits (e.g. [41]). A small fraction of animals died soon after initiation of experiments (less than 5%) and were quickly replaced to maintain treatment densities.

### Competition Experiments in the Field: *F. crassa* and *S. lessoni*

In the mid-intertidal zone of wave exposed platforms inside the marine reserve of ECIM we selected fifteen 25×25 cm arenas which hosted a central crevice of intermediate size (average size  = 20 cm long × 3.0 cm wide × 3 cm deep). To enclose individuals inside the experimental arenas, we used stainless steel hardware cloth fences (8 cm high, 10 mm mesh size) fastened to the rock with stainless steel screws. Gaps between the substratum and the base of the fence were sealed with plastic mesh. To exclude other grazers and predators from the enclosures, we painted sides and corners of the fences with antifouling copper paint. To evaluate intraspecific effects, we enclosed con-specific individuals at either “average natural densities” (6 individuals/arena for *S. lessoni*, 2 for *F. crassa*), or at high densities (12 individuals/arena for *S. lessoni*, 4 for *F. crassa*) (Table 2). To evaluate interspecific effects we used mixed-species cultures at average densities observed in the field (8 individuals per arena, see Table 2). Treatments were randomly allocated to experimental areas and replicated three times. Mixed species treatment at high densities proved difficult to maintain in the field (individuals escaped) and therefore they were not included in analyses (see Table 2). Similarly, maintenance of 8 *F. crassa* within plots was impossible because of the territorial behaviour of this species, and therefore, the design could not estimate intraspecific effects at all experimental densities [33], [42].

**Table 2:** Treatments used in field experiments, number of limpets per enclosure and average individual body size (wet weight in g).

Treatments	Limpets in enclosure	Individual Biomass ± SE (g)
*Intraspecific interactions*		
**S**	**6**	0.43±0.02
**F**	**2**	62.3±3.13
**S_ × 2 (increased × 2)_**	**12**	0.34±0.05
**F_× 2_**_**(increased × 2**)_	**4**	55.4±3.01
*Interspecific interactions*		
**S+F**	**6+2**	0.30±0.45+65.3±3.92

**S** = Siphonaria lessoni; **F** =  Fissurella crassa.

Individuals used in these experiments were collected from inside ECIM and from nearby areas immediately outside, ranging 0.67 to 1.15 cm for *S. lessoni* and 4.5 to 6.5 cm for *F. crassa* which are within the average size recorded for these grazers in the study site (see Fig. S1). Animals were held briefly in the laboratory where wet weight and shell length were recorded and then individually marked with a numbered bee tag. Soon after, *S. lessoni* were released into experimental enclosures during day low tide and 3 hours later, at nigh time, *F. crassa* were released in the enclosures. In this manner, animals were released into experimental arenas when they are most active and could reattach to the rock [28]. The procedure also allowed priority establishment of the smaller *S. lessoni* in crevices. The experiment was initiated June 01 and terminated in October 10, 2009. Twice a month, during seven consecutive days at day and night, we recorded the number of active i.e. moving or feeding, individuals inside and outside the crevice in each experimental area at 15 minute intervals for 3 to 4 hours. We also measured nearest neighbour (hereafter NN) distances to con- and heterospecific (for *S. lessoni* only) individuals in the different treatments. Con- and heterospecific NN-distances were measured when animals of both species were at rest which gave us information about individual segregation and aggregation patterns. At the end of the experiment we measured shell length and weighed all remaining animals. Since initial body size was similar among individuals, after one-way ANOVA comparison showed no significant differences in initial weight of individuals (for *S. lessoni*: F_2,6_ = 2.08; MS = 0.099; P = 0.205; *F. crassa*: F_2,6_ = 2.25; MS = 77.2; P = 0.186), growth rates (GR) were simply calculated as GR = (Wt -Wo)/*t*, where Wo = wet weight at the start, Wt = the wet weight at the end, and *t* = elapsed time in days. In addition, in order to monitor food availability for the focal grazers, algal cover inside the experimental arenas i.e. mainly periphyton, ulvoids and crustose algae, was quantified with a quadrat with 81 intersection points and photographed once a month (see Fig. S2).

To examine the potential effects of the experimental manipulations on individual behaviour [43], concurrently with experiments, we monitored the displacement and foraging activity of 15 “manipulated” (handled, marked and transplanted to a different place) and 15 “un-manipulated” (marked directly in the field but not handled) animals of each species outside experimental areas.

### Data Analysis

To evaluate the significance of treatment effects on growth rates (wet weight), separate nested ANOVA’s were conducted for each species included in the field experiments, using the average growth rate of all individual within an experimental arena as independent replicates, and individual growth rates of each arena as nested within experimental treatment. Data were log-transformed to improve variance homogeneity and normality after inspection of residuals. We use a Cochran’s C test to compare variances. Treatments were considered fixed with three levels: the two con-specific densities (average and high density) and the mixed-species treatment (see Table 2). In the case of significant effects, two planned contrasts were used to compare the two conspecific treatments against each other (intraspecific effects), and the average density treatment i.e. six individual/arena, against the mixed-species treatment (interspecific effects). Since these contrasts were not orthogonal, a Dunn-Sidák correction was used to adjust significance levels. Significance of treatment effects for the proportion of time individuals were observed using crevices and the total individual activity during the study in both laboratory and field experiments, were tested with separate one-way ANOVA’s and planned contrasts for each species, as described above. It must be noted that all individuals in enclosures were monitored during the study. In the case of use of crevices, the ‘long-term’ average proportion of individuals using crevices in each experimental area during daytime for *F. crassa* and night time for *S. lessoni*, throughout the study, was analyzed. Differences in the time of observation followed the natural rhythms of activity/resting in these species [28].

The statistical analyses described above provide a broad estimate of significance of treatment effects, but since mixed treatment enclosed a total number of individuals slightly different than the high density treatments of conspecifics, intra- and interspecific effects cannot be completely separated in these analyses. Moreover, interspecific contrasts evaluate both, the effect of changing density in addition to adding individuals of a different species. Therefore, to provide estimates of intra- and interspecific effects in the laboratory and field experiments (see [44] for review of other interaction strength measures), we first estimated *per capita* intra- and interspecific effects for each species on different response variables (i.e. growth rate, use of crevices, or activity). For a given species *i*, the per capita intraspecific effects (IS_i_) were calculated as:


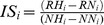
, where *RN_i_* is the *per capita* “response variable” (e.g. growth rate, use of crevices) of species *i* under the average or “natural” density treatment, *RH_i_* is the per capita response measured in the high density treatment, and *NN_i_* and *NH_i_* are the numbers of individuals in the average and high density treatments, respectively.

The total per capita inter-specific effect (*Total IS_ij_*) of species *j* on species *i* was then calculated as:


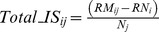
, where *RM_ij_* is the per capita response of species *i* measured in the mixed species enclosures with species *j*, and *N_j_* is the number of individuals of species *j* in those enclosures.

Calculated in this manner, per capita interspecific effects of species *j* on per capita response of species *i* estimated both, the effect of adding individuals of a different species, and the effect of changing total density of individuals inside the experimental areas (as in the ANOVA contrast). Thus, *Total IS_i_*_j_ does not separate between “pure” per capita interspecifics effects, from the expected changes observed if individuals of the same species were added to the arena (intraspecific effects). Therefore, assuming that per capita intraspecific effects would remain constant (and linear over that density range) in the presence of heterospecifics, we obtained an estimate of pure per capita *IS_ij_* as 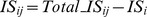.

Confidence intervals for estimates of per capita interaction strengths were obtained through bootstrapping procedures [45]. We then evaluated whether the 95% bootstrapped confidence intervals overlapped zero to judge if the particular effect was statistically significant.

For NN-distances among conspecific *S. lessoni* and *C. granosus* in the laboratory experiment, and *F. crassa* in the field experiment we calculated the *R* index proposed by Clarke & Evans [46], which determined whether distributions were aggregated, uniform or random in the conspecific density treatments and in mixed-species cultures. The index is calculated as: *R = ∑r/N×2√ρ* where *r* is the distance to nearest neighbour, *N* is the number of individuals and *ρ* is the density considered in the enclosures. In order to reduce “reflexive” nearest neighbour pairs [47], we only consider a random sample of 50% of the total NN-distances recorded in each plot. This index was not calculated for *C. granosus* or *F. crassa* because of low number of individuals per arena. Significance testing of heterospecifics NN-distances (*S. lessoni* to chitons or *F.crassa*) is complex due to the dependence on species distributional patterns, therefore we only visually compared the distribution of heterospecific nearest neighbours distances with those obtained for con-specifics [46].

## Results

### Interspecific Encounters of Grazers in the Laboratory

Two to three hours after introduction of conspecific animals into experimental arenas, 55.6% of *S. lessoni* individuals found the crevices. In contrast, 90% of *F. crassa* individuals in conspecific trials found crevices after 10–15 minutes from introduction. In the case of C. *granosus,* 75% individuals found crevices after 12–15 minutes and rested in aggregations inside crevices and performed nocturnal excursions. Few animals (<8%) died in the course of the trials.

No changes in individual behaviour were observed in heterospecific encounters between *S. lessoni* and chitons. When encountering a chiton, the pulmonate limpets tended to move up onto the chitons and graze on their shells. Vice versa, when chitons were moving or foraging, they were unaltered by contact with resting or active *S. lessoni* (see Table 3). In contrast, in encounters between *S. lessoni* and *F. crassa,* we observed *S. lessoni* commonly ceased activity and clamped their shells down to the rock when in contact with *F. crassa* (Table 3). When the larger keyhole limpets were moving and encounter a pulmonate limpet, they frequently (45%, see Table 3) moved up and over the smaller *S. lessoni* individuals in a pushing-like movement, without altering the direction of movement.

**Table 3:** Percentage of individuals (%) observed to perform different behavioural responses to interspecific “encounters” in outdoor aquaria at ECIM.

Responsive Behaviour	S → C	S → F	C → S	C → F	F → S	F → C
*Clumping to the rock*	0	57	0	0	0	0
*Halting displacements*	0	3	0	5	2	5
*Aggressiveness*	0	0	0	0	45	13
*Change angle of orientation*	13	40	55	40	5	3
*No change*	87	0	45	55	48	79

Arrows indicate the heterospecific with which target species encounters.

**S** = *Siphonaria lessoni*; **F** =  *Fissurella crassa*; **C** = *Chiton granosus*.

### Competition Experiments in the Laboratory: *C. granosus* and *S. lessoni*

#### Patterns of activity

**Figure 1:**
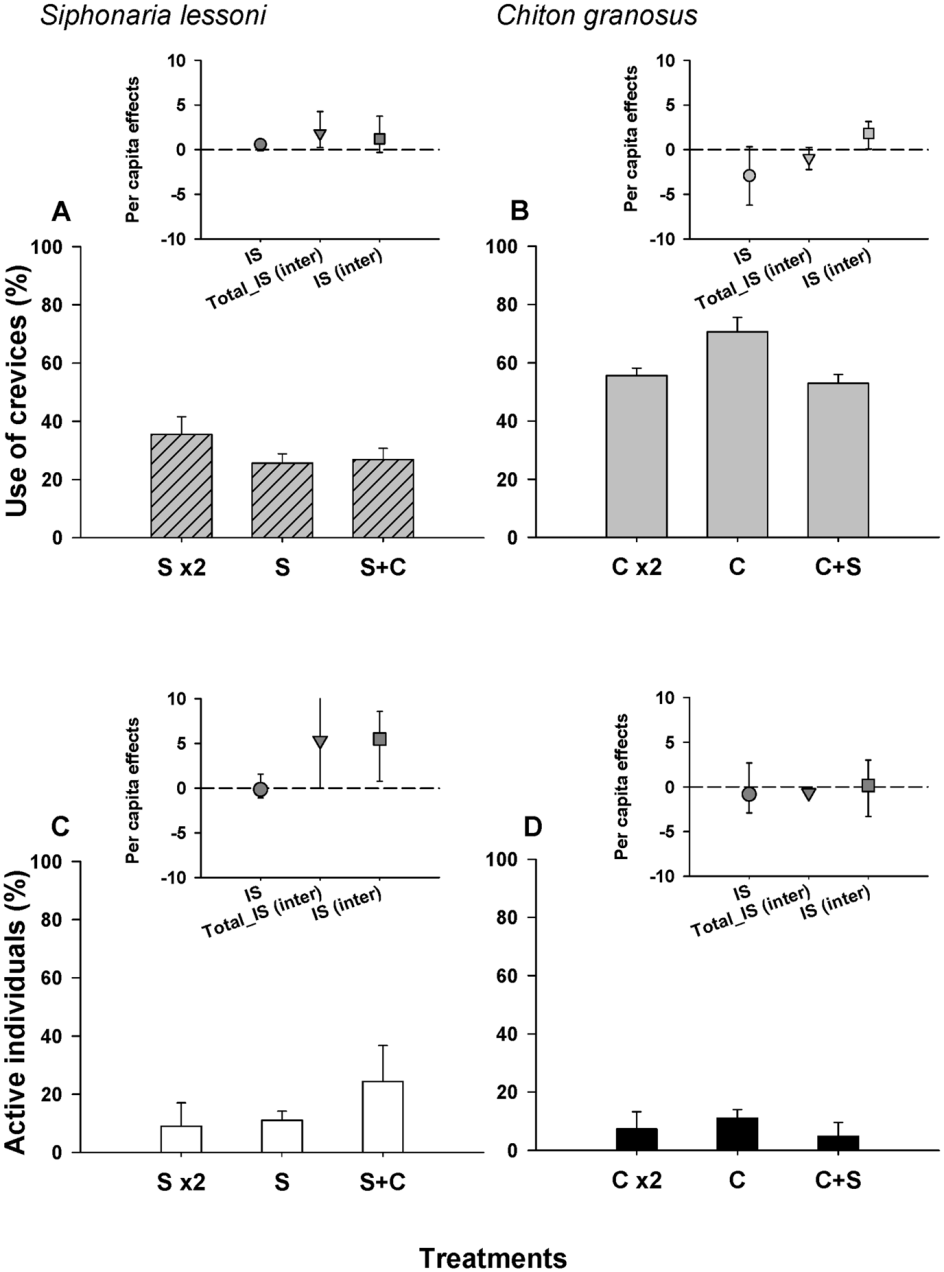
Intra- and interspecific effect responses of focal grazers under laboratory conditions. (a, b) Percentage of animals that utilized crevices and (c, d) Total active animals (%) observed in the experimental arenas during day and night. **C**: 3 *Chiton granosus*, **Cx2**: 6 *C. granosus*, **S**: 9 *S. lessoni*; **Sx2**: 18 *S. lessoni*, **S+C**: 9 *S. lessoni* +3 *C. granosus*. Inserts correspond to per capita intraspecific interaction strength (IS) and total (Total_IS (inter)) and ‘pure’ interspecific effects (IS(inter)) on the corresponding focal species for each measured variable (a and b). Bars correspond to confidence intervals (95%) estimated through bootstrapping procedure.

Surface temperature of experimental “arenas” (cement blocks) peaked between 38°C and 42°C in mid-day during simulated summer low tides, which is slightly below maximum rock surface temperatures recorded in the field at Las Cruces [48]. At the same time of the day, surface temperature inside crevices fluctuated between 17°C and 23°C. At the time of introducing animals to the experimental arenas, under immersion, rock surface temperature had equilibrated with water temperature at about 17°C. Mortality after the initial 48 h was nil in all treatments. During the 5 weeks duration of this experiment, the ANOVA analysis found no evidence of negative effects of increased intraspecific density on the allocation of time inside crevices in chitons or pulmonate limpets (Fig 1a and b), nor did we observe significant interspecific effects of *C. granosus* on the use of crevices by *S. lessoni*, or vice versa (*S. lessoni*; one-way ANOVA: F_2,6_ = 0.47; MS = 0.0574; P = 0.648; *C. granosus*: F_2,6_ = 2.95; MS = 0.2135; P = 0.1279, Fig. 1a and b). Estimates of the total *per capita* interspecific effects of *C. granosus* on *S. lessoni* showed a slightly negative, significant effect of the presence of chitons (Total_IS (inter) in insert of Fig. 1b), which became non- significant when calculating the “pure” interspecific component.

The proportion of time individuals of *S. lessoni* were active (foraging, displacement) was only slightly reduced when doubling conspecific density (Fig. 1c). In contrast, adding individuals of *C. granosus* increased average individual activity of *S. lessoni* by twofold (Fig. 1c), which was also corroborated in the significant one-way ANOVA comparison (F_2,6_ = 7.56; MS = 0.1194; P = 0.0229). Estimates of per capita effects and bootstrapped CI’s captured well the interspecific, positive effect of *C. granosus* on *S. lessoni* individual activity (Fig. 1c insert), which was significantly different from zero after correcting by changes in density (IS(inter)) (Fig. 1c insert). Diel activity rhythms of *S. lessoni* were mostly diurnal with few individuals (<10% when with conspecifics) still active a few hours after sunset (see Fig. S3a). This diel activity pattern was not affected by con- or heterospecific treatments. In the case of *C. granosus*, the proportion of individuals that were observed active in experimental arenas was unaltered by the addition of conspecifics or by *S. lessoni* (Fig. 1d, one-way ANOVA: F_2,6_ = 0.63; MS = 0.0027; P = 0.5659) and, therefore, none of the per capita effect sizes were different from zero (Fig. 1d, insert). Moreover, diel activity pattern remained strictly nocturnal, regardless of the density of con- or heterospecifics in the experimental plots (Fig. S3b).

#### Micro-spatial distribution

**Figure 2:**
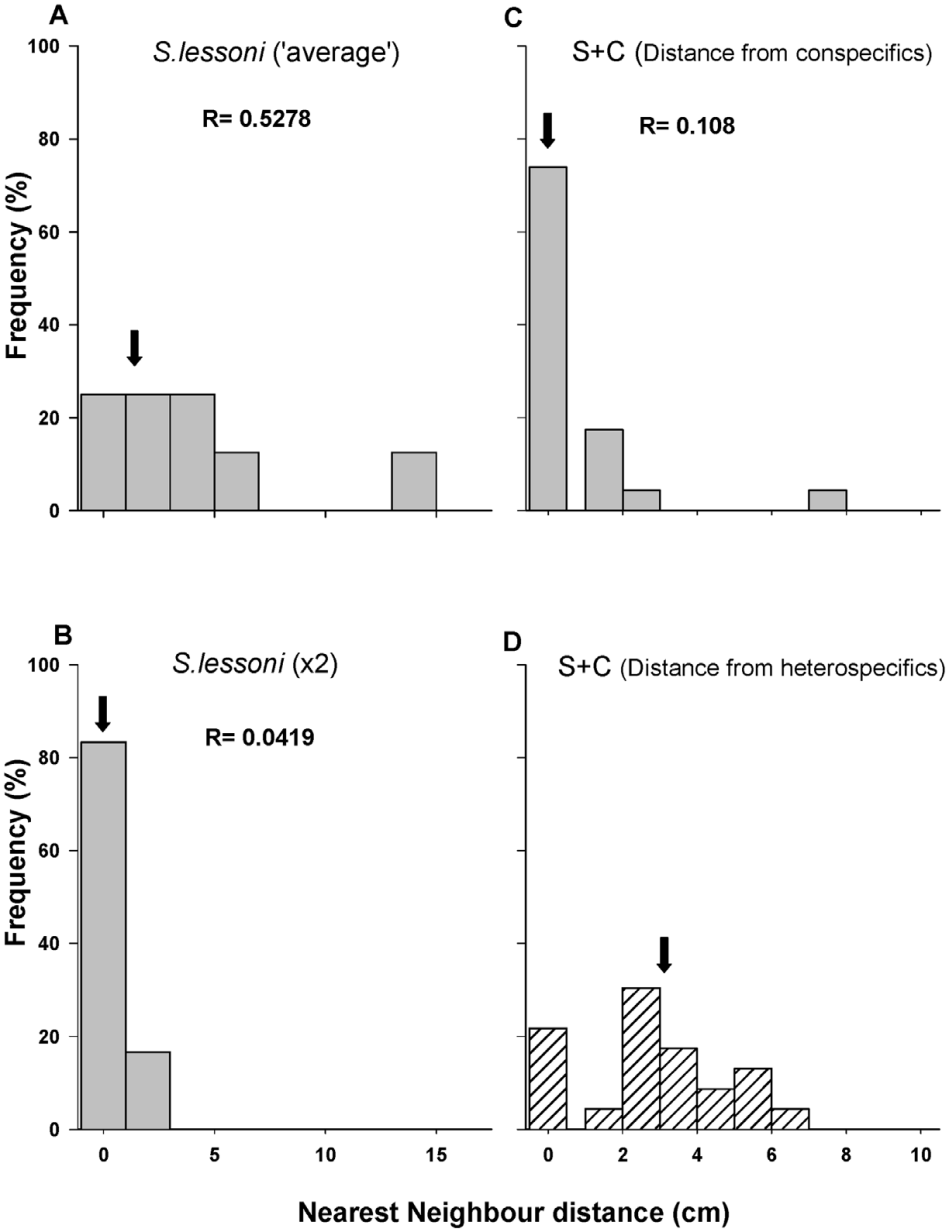
Nearest neighbor linear distances histogram for *Siphonaria lessoni*, recorded in laboratory experiments. Distances to conspecifics (a-c) and heterospecific (d) in intraspecific enclosure treatments at ‘average’ (a) and double density (b), and in interspecific (c, d) enclosure treatments (i.e. distance from *C. granosus*). Arrows show the median value. The R statistic estimate if the distributional pattern is dispersive (R = 2.0), aggregated (R = 0.0) or uniform (R = 1.0).

The median linear distances between nearest conspecific neighbours (NN) of *S. lessoni,* measured during resting decreased from 2.0 cm when alone in the ‘average density’ enclosures, to 0.0 cm in the presence of *C. granosus* (Figs. 2a and c). Similarly, doubling the density of conspecifics reduced median distance to zero and the R index, which considers the changes in density, showed an even tighter aggregated distribution (Fig. 2b). The median nearest neighbour distances between *S. lessoni* individuals and chitons (2.3 cm), measured in mixed treatments, was similar to the one between conspecifics in the ‘average density’ treatment (2.0 cm) (Figs 2a and d), suggesting that *S. lessoni* individuals do not segregate from chitons within experimental arenas.

### Competition Experiments in the Field: *F. crassa* and *S. lessoni*

#### Growth rates

**Figure 3:**
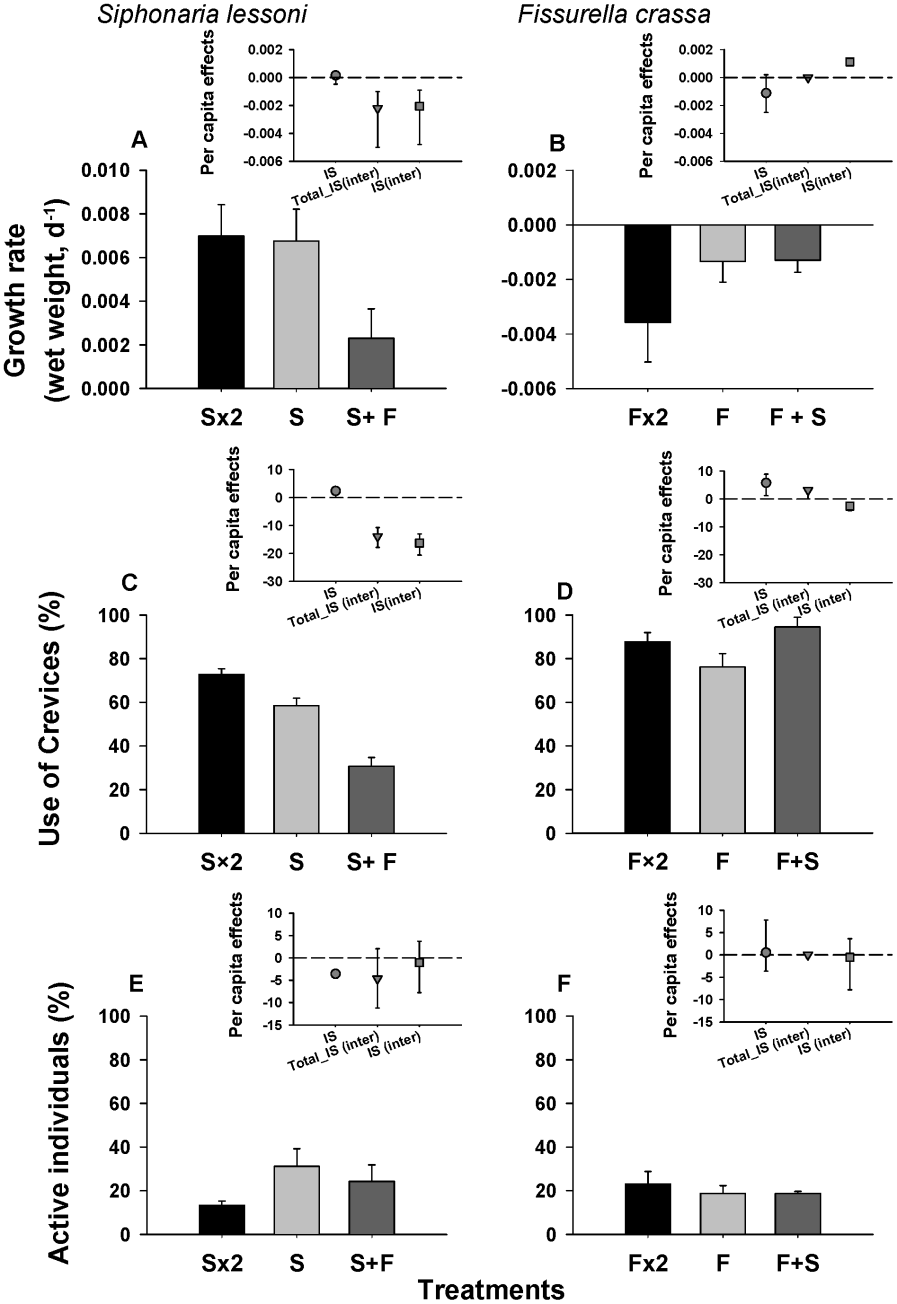
Intra- and interspecific effect responses of focal grazers in field experiments. Average (±SE) growth rate, crevice use and activity of *S. lessoni* (a-d) and *F. crassa* (e-h) recorded inside experimental arenas. **F**: 2 *F. crassa*, **Fx2**: 4 *F. crassa*, **S**: 6 *S. lessoni*, **Sx2**: 12 *S. lessoni*, **S+F** or **F+S**: 6 *S. lessoni* and 2 *F. crassa*. Inserts correspond to per capita intraspecific effects (IS), and the total (Total_IS(inter)) and ‘pure’ interspecific effects (IS(inter)) of heterospecifics on the corresponding focal species. Bars correspond to confidence intervals (95%) estimated through bootstrapping procedure.

At the end of the 125 days duration of the field experiment, significant differences in *S. lessoni* growth rates were observed among treatments (Fig. 3a, Table 4a). Planned contrasts showed that increasing density of conspecifics had no effect on *S. lessoni* growth rate, while introducing 2 individuals of *F. crassa* had highly significant negative effects (Fig 3a, Table 4a). No significant variation in growth rates were observed among arenas within treatments (Table 4a). Overall, *F. crassa* individuals lost weight during the course of the experiment (Fig. 3b) and these rates remained virtually unaltered when in the presence of *S. lessoni* (Fig. 3b). Doubling the density of conspecifics reduced growth rates by twofold, (Fig 3b), but these differences were not significant in the ANOVA (Table 4b).

**Table 4:** Nested ANOVA on average growth rate of individuals present in each arena under the different treatments in field experiments.

Source	DF	MS	F	P
**a)** *Siphonaria lessoni*				
Treatment	2	0.000072	22.5	**0.0017**
Arena (Treatment)	6	0.000003	0.15	0.9882
Residual	22	0.000022		
**Contrasts**				
Intraspecific effects (S versus S×2)	1	0.0000002	0.07	0.8065
Interspecific effects (S versus S+F)	1	0.000097	29.87	**0.0016**
**b)** *Fissurella crassa*				
Treatment	2	0.177949	1.85	0.2363
Arena (Treatment)	6	0.096057	0.75	0.6210
Residual	12	0.128013		

See Table 2 for clarification of abbreviations.

Since planned contrasts are not orthogonal, p-values were adjusted using the Dunn-Sidák correction. Significant values at α<0.05 are presented in bold face.

In agreement with significance ANOVA tests, the magnitude of *per capita* intra- and interspecific effects were strikingly different in *F. crassa* and *S. lessoni* (Figs 3 a and b inserts). The effect of *F. crassa* on *per capita* growth rate of *S. lessoni* (Total_IS_ij_) was 13 times larger than the per capita effect of *S. lessoni* on *F. crassa* (which was no different from zero, Figs 3a and b inserts). Adjusting for the net changes in density to obtain a ‘pure’ estimate of interspecific effects (IS_ij_(inter)) showed that the pulmonate limpet had a slightly positive effect on growth of *F. crassa* (Fig. 3b insert).

#### Patterns of activity

In the presence of *F. crassa*, *S. lessoni* utilized the crevices less frequently (30.6%) than when they were alone (Fig. 3c, Table 5a, interspecific contrast). Instead, increasing density of conspecifics had a slight positive effect on the use of crevices by the pulmonate limpet (Fig. 3c, Table 5a, intraspecific contrast). In contrast, *F. crassa* utilized crevices in similar proportion in both intra- and interspecific treatments (Fig. 3d, Table 5b). Per capita intraspecific effects of *S. lessoni* on use of crevices were slightly positive; while the interspecific effect of *F. crassa* was strongly negative (Fig. 3c insert), especially after correcting by the change in density. In the case of *F. crassa* there also was a positive intraspecific effect on crevice use. The total interspecific *per capita* effect (Total_IS _(inter)_) of *S. lessoni* on *F. crassa* was slightly positive, but not different from zero (Fig. 3d insert). Correcting by changes in density led to a slightly negative and significant effect of the pulmonate on use of crevice by the keyhole limpet (IS _(inter),_ Fig. 3d insert).

**Table 5:** One-way ANOVA on proportion of animals using crevices under the different treatments used in field experiments.

Source	DF	MS	F	P
**a)** *Siphonaria lessoni*				
Treatment	2	0.2259	10.30	**0.0115**
Residual	6	0.0219		
**Contrasts**				
Intraspecific effects (S versus S×2)	1	0.0328	1.50	0.2669
Interspecific effects (S versus S+F)	1	0.2209	10.08	**0.0192**
**b)** *Fissurella crassa*				
Treatment	2	0.0259	1.23	0.3561
Residual	6	0.0211		

See Table 2 for clarification of abbreviations.

Since planned contrasts are not orthogonal, p-values were adjusted using the Dunn-Sidák correction. Significant values at α<0.05 are presented in bold face.

In general, enclosed *S. lessoni* in experimental plots maintained similar levels of activity as free animals in nearby areas, suggesting that enclosures did not alter their behaviour. Increasing the density of pulmonate limpets reduced in half the proportion of animals found active (Fig. 3e) and differences were statistically significant after one-way ANOVA (F_2, 6_ = 6.08; MS = 0.0061; P = 0.0360). Estimates of per capita effects showed this negative intra-specific effect (Fig. 3e insert). The presence of *F. crassa* did not alter total activity of the pulmonate limpet (Fig. 3e) and per capita estimates did not differ from zero, indicating that the presence of *F. crassa* has no effect on levels of activity of *S. lessoni.* The pattern of diel activity in *S. lessoni* was not altered by the treatments either, i.e. individuals maintained the diurnal-activity nocturnal-resting cycle, regardless of the treatment (see Fig. S3c).

Increased density of *F. crassa* had a slightly positive, non significant effect on the levels of activity of conspecific individuals (Fig. 3f, one-way ANOVA: F_2, 6_ = 0.02; MS = 0.0001; P = 0.9844). The presence of *S. lessoni* did not alter the activity of *F. crassa* individuals (Fig. 3f). Accordingly, all estimates of intra- and interspecific per capita effects on activity of *F. crassa* were not different from zero (Fig. 3f insert). The pattern of strictly nocturnal activity was maintained in *F. crassa*, regardless of the intra or interspecific treatments (see Fig. S3d).

#### Micro-spatial distribution

**Figure 4:**
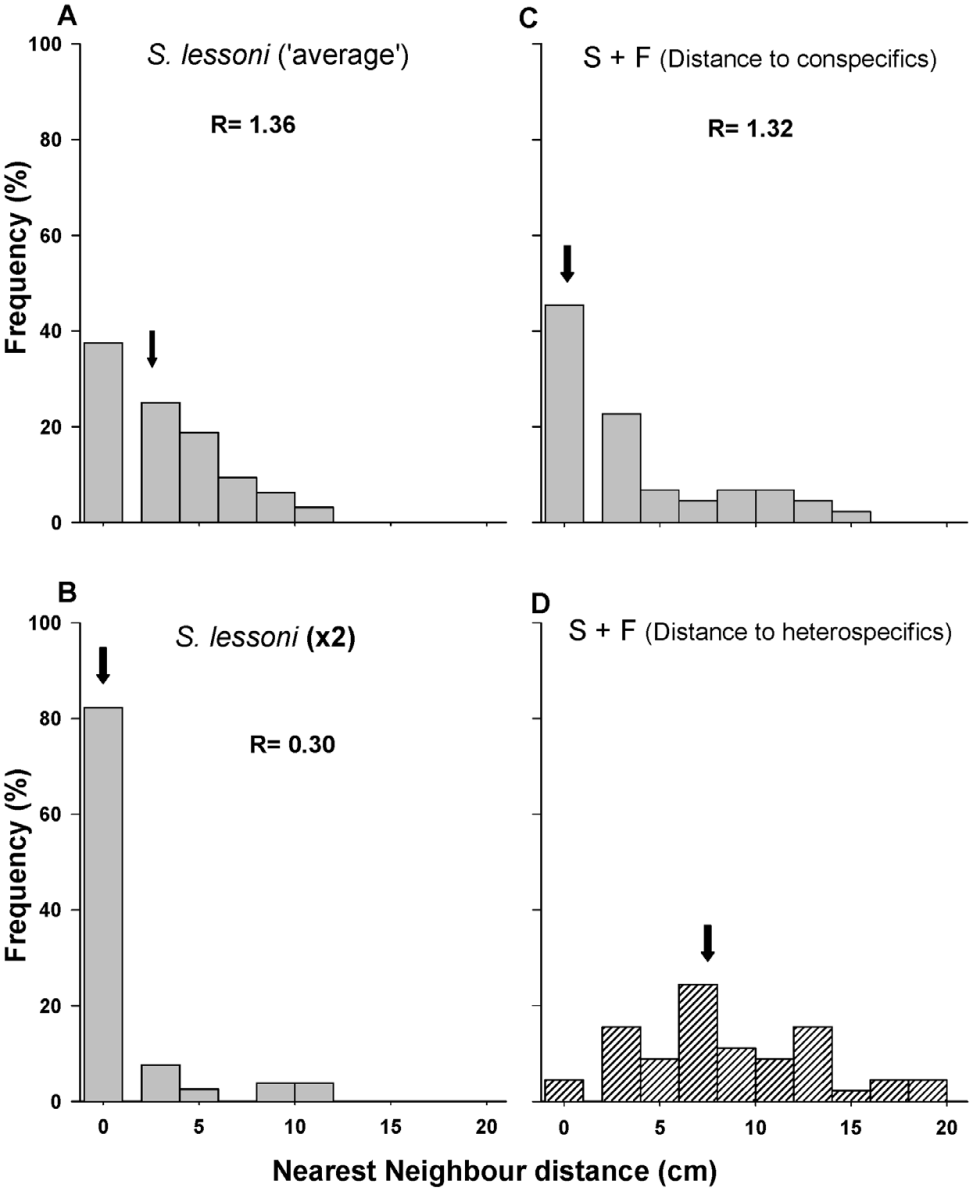
Nearest neighbor linear distances histogram for *Siphonaria lessoni* recorded in field experiments. Distances to conspecifics (a-c) and heterospecifics (d), in intraspecific enclosure treatments at natural (a) and double density (b), and in interspecific (c, d) enclosure treatments (i.e. distance from *F. crassa*). Arrows show the median value.

The median linear distances between nearest conspecific neighbours of *S. lessoni* as well as the R indices were similar in the average density treatment and in the presence of *F. crassa* (1.20 cm and 3. 44 cm respectively, Fig. 4a,c), suggesting that the keyhole limpet has no effects on aggregation behaviour of the smaller pulmonate limpet. Instead, as shown in laboratory experiments, increasing conspecific density of *S. lessoni* reduced median distances to 1.16cm and the R-index showed an even tighter aggregated distribution (Fig. 4b). While the level of aggregation to conspecifics was unaltered by the presence of *F. crassa*, individuals found places far from the keyhole limpet, conforming to a ‘repellent’ distribution of heterospecific distances (median linear distance to heterospecifics: 6.56 cm, Fig. 4d).

## Discussion

Through field and laboratory experiments, we were able to link territorial behaviour of competitively dominant *F. crassa* to alteration in spatial distribution and crevice use of the smaller and gregarious *S. lessoni*. Support for the hypothesis that spatial segregation and use of crevices is directly related to body size differences was only partial since we found no effects of competition between the smaller *S. lessoni* and the gregarious chiton *C. granosus*. Moreover, while levels of activity and use of crevices were altered by intra- and interspecific interactions, differences in diel activity patterns between *S. lessoni* and the other grazers were unaltered. Our results thus illustrate the complex structure of interactions that can take place within consumer guilds, which can be played at for non-food resources and yet might modulate their functional roles on lower trophic levels. On the one hand, the small-scale spatial segregation observed in the field between two gregarious species, *S. lessoni* and *C. granosus* seem to result from slight, species-specific differences in habitat use and/or similar gregarious behaviour which might compensate for size differences as we found no evidence of interspecific competition. On the other hand, we present strong evidence that spatial segregation between the large territorial keyhole limpet *F. crassa* and the smaller *S. lessoni* results from highly asymmetric competition which is related to interference in the use of shelters, rather than exploitation competition for food. The presence of *F. crassa* had strong negative effects on the use of crevices and growth rates of *S. lessoni*, while the smaller limpet had variable effects (i.e. weakly negative to positive) on the keyhole limpet. Thus, gregarious behaviour did not compensate for larger body size, and diel differences in activity rhythms were not sufficient to prevent competition for shelters. Here we discuss these findings and highlight the importance of interference competition in this herbivore assemblage.

### Interference for Shelters Versus Exploitation of Food

In our system, the focal herbivore species overlap amply in diet, generating the potential of interspecific exploitative competition, as observed, for instance, among gastropod species [41] as well as within limpet species [49] on South East Australia. However, the generalist diets of Chilean grazer species, composed mostly by microalgae (‘biofilm’), non-calcareous crusts, germlings of green ephemeral algae and even recently settled stages of sessile and mobile invertebrates [17], [29], [36], [37], together with the generally high productivity of Chilean coastal ecosystems [38], [39], reduces the likelihood that food limitation could have significant effects on individual performance. Moreover, as succession progresses, plants become established and ephemerals are replaced by corticated algae [50], creating opportunities for differentiation in feeding sources, as shown recently between *Fissurella* and the other intertidal herbivore species [17]. Therefore, although rigorous experiments controlling resource productivity are lacking, it is unlikely that under the conditions encountered in central Chile exploitative competition for food plays a major role in the structure of the herbivore assemblage. Inside the experimental arenas used in our field and laboratory experiments, microalgae, ulvoids and non-calcareous crusts (only in the field), were abundant throughout the study (see Fig. S2), suggesting that food was not a limiting factor during experiments. Instead, interference and/or exploitative competition for shelters can be important for those species which need shelter during resting periods to avoid predators or to reduce desiccation and temperature stresses, as we show here for *F. crassa* and *S. lessoni*. Fast occupation of the crevices and/or direct interference by *F. crassa* i.e. pushing-like movements at encounters with *S. lessoni* as seen in laboratory, could account for low use of these shelters by *S. lessoni*. Whichever the mechanism, intra- and interspecific competition for shelters may help limit local population density to levels below those needed to overexploit food resources and, therefore, be of chief importance in the structure of the consumer guild. Indeed, in field experiments and despite algae were common in the experimental arenas, increasing conspecific density of the dominant *F. crassa* led to sharp decrease in growth rates, which can be interpreted as an indication that crevices are a limiting factor.

The importance of exploitative competition for food is expected to vary across regions with different productivity or different availability of palatable algae [e.g. 51]. This might be the case between central and southern Chile. Indeed, studies conducted in southern Chile suggest that growth rates and size structure of *S. lessoni* are negatively affected by exploitative competition for food with the large fissurellid *Fissurella picta* [52]. But further experimental studies controlling food renewal rates (e.g. [53]) and availability of shelters [54] would be necessary to draw conclusions about the importance of spatial variation in food limitation for these limpets.

Despite differences in size the gregarious chiton *C. granosus* had no measurable effects on *S. lessoni*, except for an increase in overall activity of individuals, but that did not alter either the use of crevices, diel activity rhythms or spatial distribution. It is unclear which could be the consequences of increased individual activity since there is no evidence of negative/positive behavioural encounters, and the short duration of the experiment did not allow us to evaluate growth rates.

### Use of Shelters; Individual and Population Effects

Several studies have shown that shelters, like rock crevices, are suitable habitat for different intertidal grazer and carnivore species, determining patterns of population density and influencing spatial patterns of foraging behaviour [27], [54]. Availability of crevices could diversify spatial niches that can be exploited by different species constituting key resources for grazers. Interference and even exploitative competition for shelters is expected to be common in grazer species, especially among those subjected to intense levels of predation (e.g. [15], [25]) or desiccation and temperature stresses (e.g. [27]), as well as among territorial species [8], [55]. We believe that the observed reduction in growth rate of *S. lessoni* in the presence of *F. crassa* results from the increased time spent outside crevices during daytime low tides, which likely increased desiccation of individuals, energy expenditure (e.g. [31]) and possibly produced reduced feeding rates, although no alteration in individual activity levels was observed in response to *F. crassa*. In line with this explanation, increased conspecific density of *S. lessoni* led to a slight non significant increase time spent inside crevices and to no effects on growth rates, i.e. no measurable intraspecific competition with a doubling in *S. lessoni* density. Although *S. lessoni* aggregated more tightly at higher than at lower density of both con- and heterospecifics (*F. crassa* and *C. granosus*), reduction in growth rates is more likely attributed to reduced use of crevices rather than to changes in aggregation of individuals. It is unclear why *S. lessoni* appear to be less gregarious at lower densities (after compensating for overall density). It may be related to limited mucus trails and poor orientation of individuals [56]. But in any case, increased aggregation in gastropods has been frequently associated to reduction in body temperature [57], which should lead to increased rather than decreased growth rates. In other cases, however, aggregation behaviour has no effects on desiccation or temperature stresses (see [58], [59] for discussion).

Since our experiments were designed to evaluate competition, we excluded predators, except for the occasional bird that could feed through the top of the fence (not seen). Therefore we cannot assess the importance of predation in shelter utilization by grazers. Spatial gregarious distribution and availability of shelters has been observed to reduce predation risk in terrestrial and marine herbivores (e.g. [9], [60]). For example, in tropical systems it has been observed that predation risk increases in areas near or just outside shelters of damselfishes (i.e. branching corals and anemones) [9]. The same study showed that predation risk faced by damselfish was increased by intraspecific interference competition for these microhabitats [9]. Thus incorporation of mortality risks due to visual predators (mainly birds and fish) would certainly intensify the consequences of competition for shelters among our focal grazers.

Inside Las Cruces marine reserve, densities of both *S. lessoni* and *F. crassa* were higher than in open access shores, while body size of *S. lessoni* was smaller and that of *F. crassa* larger inside marine reserve as compared with adjacent platforms (see Fig. S1). It is possible that exploitation of rock crevices by large *F. crassa* inside the marine reserve impedes the use of the best shelters by *S. lessoni*, whose individuals were commonly observed resting inside empty barnacle shells not accessible to keyhole limpets. As of yet, it is unclear whether different grazers exhibit active preferences for different types of shelters or which is the size-related availability of shelters within the reserve or outside. Future studies should take into account shelter availability in different systems as relevant habitats for population persistence, because in face of increased spatial alteration of ecosystems their distribution could have profound consequences for the structure of consumer assemblages (e.g. [61]).

Patterns of daily activity or rhythms as well as the total time individuals are active (searching, foraging, mating) are usually endogenous, but modulated by environmental factors [30], [62]. In our experiments none of the species changed diel activity rhythms in response to changes in density or the presence of heterospecifics (Fig. S3), suggesting such rhythms are fixed.

### Individual Density, Size and Experimental Limitations

A major challenge when designing experimental studies is to manipulate density but keep all other attributes of species, such as spatial distribution and behaviour as ‘natural’ as possible within experimental areas. The combination of different spatial patterns of distribution, (from territorial to highly gregarious), foraging behaviour (from extreme site fidelity to dispersive foraging) and widely different body sizes [28], made it impractical to maintain the density levels necessary to generate a “complete” competition design (e.g. [41]) within reasonably sized experimental arenas. However, under the assumption that per capita intraspecific effects do not vary dramatically with the presence of heterospecifics and with small changes in conspecific density, we provide estimates of intra- and interspecific competition effects that agree well with our behavioural observations in the laboratory.

Alteration of individual behaviour due to enclosures seem to have been kept to a minimum as animals did not show altered movement patterns with respect to unmarked individuals [17]. Yet, the confinement of heterospecifics within the fences restricted the distance individuals could segregate either from each other in the case of *F. crassa*, or between *S. lessoni* and *F crassa*, especially while foraging [28]. Therefore, absolute nearest neighbour distances should be interpreted with caution. Furthermore, confinements of two large (6.2±0.12 cm average shell length) *F. crassa* individuals constrained their large foraging excursions (>50 cm long), although they did not alter the typically strong site fidelity, compared with un-manipulated animals recorded in the same shores in a previous study [28].

Our study illustrate the importance of interference competition for shelters between species of intertidal herbivores, which results in behavioural changes of the smaller-sized species, and how these fast plastic responses can translate into differences in growth rates of individuals. This further highlights that modifications of behavioural traits could have direct consequences on the spatial structure of the assemblage. Shelter use behaviour and interspecific individual distribution at resting and foraging seems plastic responses to the presence of competitors. When evaluating the functional structure of consumer guilds, including species specific roles and levels of complementarity or redundancy, researchers often examine interactions that take place through food resources. We show that other limiting resources, such as the availability of shelters, can impose constraints that can be at least as important on consumer guild structure as the interactions that take place through food consumption. Thus, patterns in the use of shelters, which can be modulated by environmental conditions in a species-specific as well as an interactive manner within the same guild, can have direct consequences on lower trophic levels.

## Supporting Information

Figure S1 **Estimation of individual traits of** ***Siphonaria lessoni*** **inside and outside the marine reserve.** Frequency histogram of shell size (mm) and wet weight (g) of the species *Siphonaria lessoni* recorded inside human protected marine reserve at ECIM, and in open access platforms at the locality of Pelancura distant aprox. 8 km south the marine reserve. Arrows indicate median values.
(TIFF)

Figure S2 **Algal abundance inside experimental areas.** Average (± SE) percent cover of the main algal groups recorded with 25×25 cm quadrat inside experimental enclosures (field experiments) through five month of study. Key for treatments: Monocultures: **F**: 2 *F. crassa*, **Fx2**: 4 *F. crassa*, **S**: 6 *S. lessoni*, **Sx2**: 12 *S. lessoni*. Mixture treatment: **S+F**: 6 *S. lessoni* and 2 *F. crassa.*
(TIF)

Figure S3 **Diel activity rhythms of focal grazers.** Average (± SE) percentage active individuals found to be active during (a, b) laboratory experiments (i.e. inside cement blocks) and during (c, d) field experiments at day (white bars) and night (black bars). Key for treatments: laboratory: **C**: 3 *Chiton granosus*, **Cx2**: 6 *C. granosus*, **S**: *9S. lessoni*; **Sx2**: 18 *S. lessoni*, **S+C**: 9 *S.lessoni* +3 *C.granosus*. Field: **F**: 2 *F. crassa*, **Fx2**: 4 *F. crassa*, **S**: 6 *S. lessoni*, **Sx2**: 12 *S. lessoni*, **S+F**: 6 *S. lessoni* and 2 *F. crassa*.
(TIF)
