# Supplementary figures and images for: Interspecific Competition for Shelters in Territorial and Gregarious Intertidal Grazers: Consequences for Individual Behaviour
Source: PLoS One. 2012 Sep 25;7(9):e46205. doi: 10.1371/journal.pone.0046205 (PMC3458018; doi:10.1371/journal.pone.0046205)

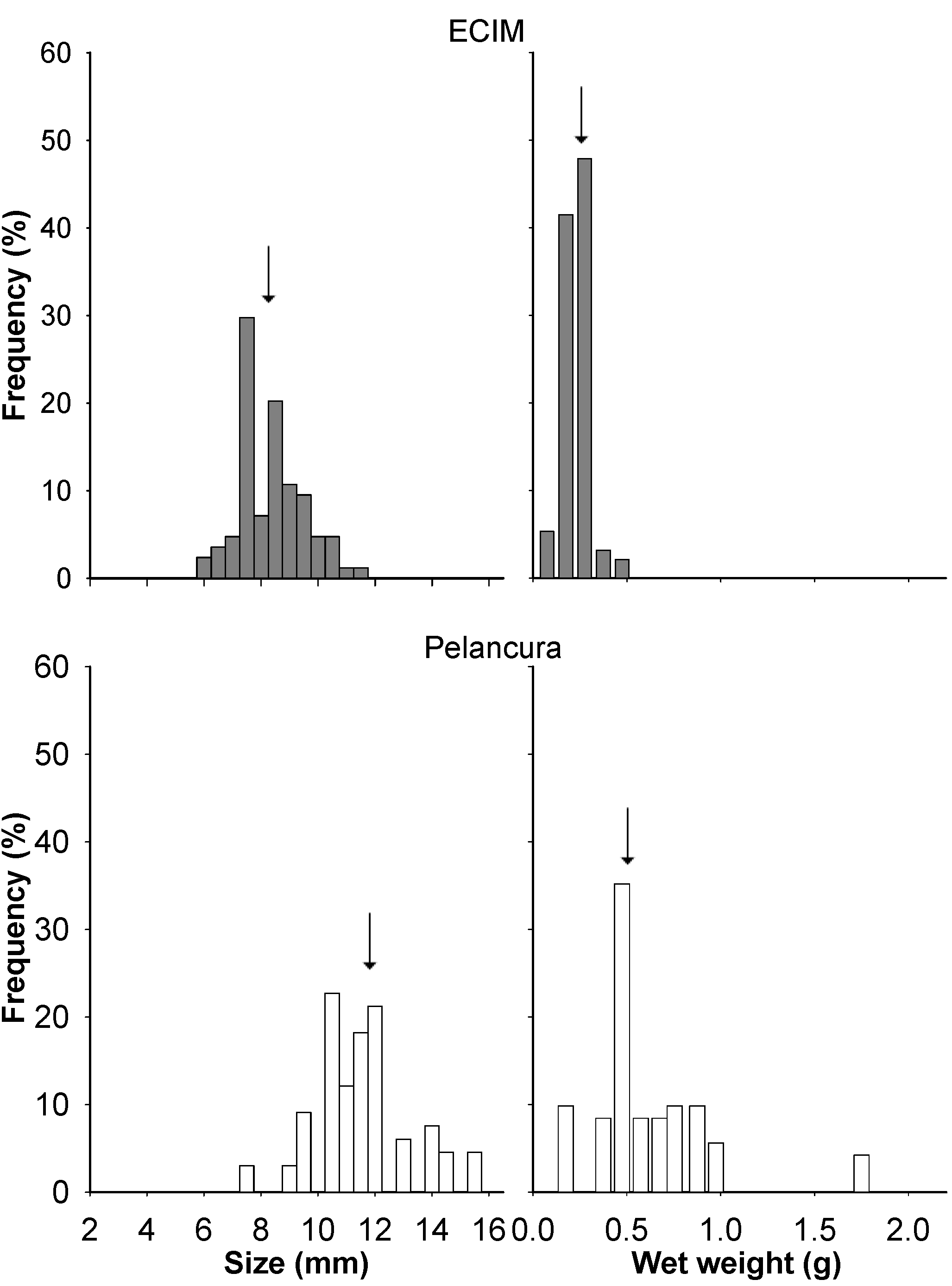

Supplement: Figure S1 — Estimation of individual traits of Siphonaria lessoni inside and outside the marine reserve. Frequency histogram of shell size (mm) and wet weight (g) of the species Siphonaria lessoni recorded inside human protected marine reserve at ECIM, and in open access platforms at the locality of Pelancura distant aprox. 8 km south the marine reserve. Arrows indicate median values. (TIFF) [file pone.0046205.s001.tiff]

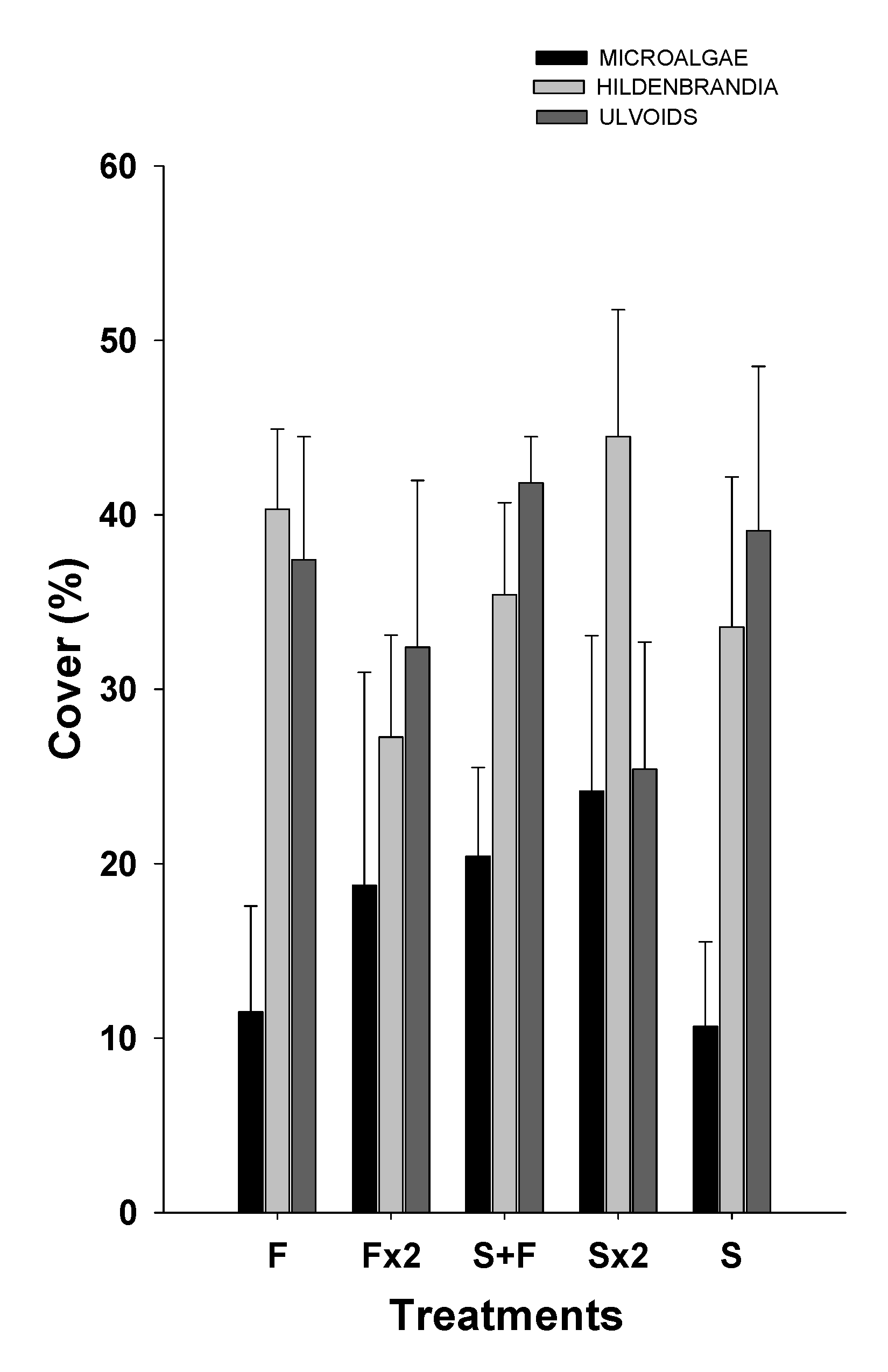

Supplement: Figure S2 — Algal abundance inside experimental areas. Average (± SE) percent cover of the main algal groups recorded with 25×25 cm quadrat inside experimental enclosures (field experiments) through five month of study. Key for treatments: Monocultures: F: 2 F. crassa, Fx2: 4 F. crassa, S: 6 S. lessoni, Sx2: 12 S. lessoni. Mixture treatment: S+F: 6 S. lessoni and 2 F. crassa. (TIF) [file pone.0046205.s002.tif]
